# Health Outcomes Linkage in UK Biobank

**DOI:** 10.23889/ijpds.v9i5.2771

**Published:** 2024-09-18

**Authors:** Laura Bramley, Jo Gay, Christopher de Lacy, Howard Callen, Sean Rose, Amy Flaxman, Gemma Codner, Rachael Drake-Brockman, Ben Lacey, Naomi Allen

**Affiliations:** 1 Nuffield Department of Population Health, University of Oxford; 2 UK Biobank

UK Biobank is a biomedical database containing de-identified data for over 500,000 participants within the United Kingdom, made globally available to researchers for health-related research in the public interest. To obtain comprehensive health outcome data, UK Biobank links to participants’ electronic medical record (EHR) data from the National Health Service (NHS) amongst others. This currently involves fifteen data pipelines covering death and cancer registries, inpatient records, primary care records, and COVID-19 test and vaccination data, with numerous others planned.

Integrating diverse “real-world” datasets into the resource necessitates a complex data infrastructure and thorough quality assurance. Challenges include file format changes, linkage problems, incomplete or invalid records, problems with encoding systems and discordances between different data sources.

Various methods can be used to integrate data into the resource, including commercial software, internally developed tools, and custom-scripted pipelines for individual feeds. To streamline and standardize data processing, we are trialing a new data integration architecture and toolset. This aims to reduce manual input, and improve transparency, quality assurance and efficiency within and between data pipelines.

The pilot is currently ongoing with results expected in mid-2024. We anticipate these new tools will help to streamline the end-to-end process, which will allow the data analysis and linkage teams to focus on further improving data quality and providing more ‘research-ready’ summary outputs on researchers’ health outcomes of interest. The findings of the pilot will be relevant to researchers and data scientists looking to employ cutting-edge approaches to linkage of any large-scale population data.

